# Characterization of coagulase-negative staphylococcal isolates from blood with reduced susceptibility to glycopeptides and therapeutic options

**DOI:** 10.1186/1471-2334-9-83

**Published:** 2009-06-04

**Authors:** Silvia Natoli, Carla Fontana, Marco Favaro, Alberto Bergamini, Gian Piero Testore, Silvia Minelli, Maria Cristina Bossa, Mauro Casapulla, Giorgia Broglio, Angela Beltrame, Laura Cudillo, Raffaella Cerretti, Francesca Leonardis

**Affiliations:** 1 Intensive Care Unit, Department of Surgery, "Tor Vergata" University of Rome, Via Montpellier 1, 00133 Rome, Italy; 2 Clinical Microbiology Laboratories, Polyclinic of Tor Vergata, Viale Oxford 81, 00133 Rome, Italy; 3 Infectious Diseases, Department of Public Health, "Tor Vergata" University of Rome, Via Montpellier 1, 00133 Rome, Italy; 4 Haematology, Department of Biopathology, Polyclinic of Tor Vergata, Viale Oxford 81, 00133 Rome, Italy; 5 Dipartimento delle Emergenze e di Accettazione, di Medicina Critica del Dolore e delle Scienze Anestesiologiche -UOSD Terapia Intensiva, Policlinico Tor Vergata, Vale Oxford 81, 00133 Roma, Italy

## Abstract

**Background:**

Coagulase-negative staphylococci (CoNS) are a major cause of nosocomial blood stream infection, especially in critically ill and haematology patients. CoNS are usually multidrug-resistant and glycopeptide antibiotics have been to date considered the drugs of choice for treatment. The aim of this study was to characterize CoNS with reduced susceptibility to glycopeptides causing blood stream infection (BSI) in critically ill and haematology patients at the University Hospital Tor Vergata, Rome, Italy, in 2007.

**Methods:**

Hospital microbiology records for transplant haematology and ICU were reviewed to identify CoNS with elevated MICs for glycopeptides, and isolates were matched to clinical records to determine whether the isolates caused a BSI. The isolates were tested for susceptibility to new drugs daptomicin and tigecycline and the genetic relationship was assessed using f-AFLP.

**Results:**

Of a total of 17,418 blood cultures, 1,609 were positive for CoNS and of these, 87 (5.4%) displayed reduced susceptibility to glycopeptides. Clinical review revealed that in 13 cases (7 in haematology and 6 in ICU), CoNS with reduced susceptibility to glycopeptides were responsible for a BSI. *Staphylococcus epidermidis *was the causative organism in 11 instances and *Staphylococcus haemolyticus *in 2. The incidence of oxacillin resistance was high (77%), although all isolates remained susceptible to linezolid, daptomycin and tigecycline. Fingerprinting of CoNS identified one clonal relationship between two isolates.

**Conclusion:**

Multi-resistant CoNS with reduced susceptibility to glycopeptides, although still relatively infrequent in our hospital, are emerging pathogens of clinical concern. Surveillance by antibiotyping with attention to multi-resistant profile, and warning to clinicians, is necessary.

## Background

Coagulase-negative staphylococci (CoNS) are normal commensals of the skin, anterior nares, and ear canals of humans. They have long been considered as nonpathogenetic, and were rarely reported to cause severe infections. However, as a result of the combination of increased use of intravascular devices and an increase in the number of hospitalized immunocompromised patients, CoNS have emerged as a major cause of nosocomial bloodstream infections [1-3].

CoNS, primarily *Staphylococcus haemolyticus *and *Staphylococcus epidermidis*, are often resistant to multiple antibiotics, and glycopeptides have been considered the drugs of choice for the management of infections caused by these organisms [4,5]. However, widespread use of glycopeptides recently has led to the emergence of CoNS isolates with decreased susceptibility to glycopeptides, displaying a minimal inhibitory concentration (MIC) of 4–8 mg/L for vancomycin and/or a MIC of 8–16 for teicoplanin [6-8]. Although acquired resistance to glycopeptides was first documented in CoNS in 1986 [9], attention was focussed on glycopeptide resistance in enterococci [10,11], due in large part to the historically relatively infrequent occurrence of CoNS infections.

In the late 1990s, the emergence of glycopeptides resistant *Staphylococcus aureus *in Japan [12] and in the USA [13] and later reports on hetero-resistance to glycopeptides in MRSA [14,15] raised the issue of the limited therapeutic options remaining for these Gram positive multi-resistant bacteria that largely cause nosocomial infections. The extensive clinical isolation of multi-resistant CoNS, especially from blood, poses similar clinical concerns and challenges.

At the University Hospital of Tor Vergata, Rome, Italy, CoNS are most frequently isolated from blood cultures taken from haematologic patients and from critically ill patients admitted to the intensive care unit (ICU). Occasionally, these CoNS isolates display a reduced susceptibility to glycopeptides. Since the majority of these isolates can be considered as contaminant, the incidence of CoNS with reduced sensitivity to glycopeptides actually causing bloodstream infections (BSI) is difficult to determine. The objective of this study was to investigate whether glycopeptide-resistant CoNS represent a significant clinical concern by examining CoNS isolates and patient records for 2007. The second aim was to determine whether the infecting bacteria were clonally related and to review the therapeutic options.

## Methods

This study was approved by local Ethical Committee (University of Rome – Polyclinic Tor Vergata) on march 2008. We reviewed University Hospital Tor Vergata laboratory records in 2007 to identify any CoNS isolated from blood having a minimal inhibitory concentration (MIC) of ≥ 2 μg/mL for vancomycin and/or an MIC of ≥ 8 μg/mL for teicoplanin. The search was limited to the transplant haematology ward and the ICU where patient and clinical data are routinely collected in a local database in which every infective event is catalogued. All clinical isolates were registered and stored at -80°C in defibrinated bovine blood. Only non-duplicate blood stream isolates included in the analysis.

We obtained information on the clinical course and outcome, microbiological outcome, any antecedent use of antimicrobial agents and actual therapy for each patient who had a blood stream infection with a CoNS with reduced susceptibility to glycopeptides.

Criteria used to categorize isolates as contaminants versus blood stream infection were the following:

1) fever or signs of sepsis at the time of blood culture;

2) isolation of the same potential skin contaminant from two or more blood cultures drawn on separate occasions within a 48-hour period AND isolated from a patient with an intravascular access device inserted at least 48 hours before AND physician institutes appropriate antimicrobial therapy;

3) absence of any other possible site of infection;

To define whether an infection was catheter-related, one of the following criteria had to be satisfied:

1) catheter tip was positive for the same agent retrieved at a peripheral vein

2) blood culture drawn from catheter hubs revealed the same agent isolated from peripheral vein but at least 120 minutes before the latter.

All the infectious events were classified by an expert infectivologist in the local database as the microbiology results were obtained.

### Laboratory methods

Biochemical identifications and antibiotic susceptibility tests were performed using the VITEK 2 automated system (bioMerieux) following the manufacturer's instructions. Susceptibility to tygeciclin and daptomycin was tested using the appropriate E-test (AB Biodisk) following the manufacturer's instructions. The strains were first induced to express glycopeptides resistance by culture on Vanco- agar screen plate, according to the Clinical and Laboratory Standards Institute (appendix B) and CDC guidelines, containing 6 μg/mL of vancomycin [16,17]. Then they were tested with authomated system as well as by E-test. The MIC (μg/mL) interpretative standard for vancomycin and teicoplanin were those suggested by EUCAST (respectively: ≤ 4 susceptible, ≥ 8 resistant).

### Amplified Fragment Length Polymorphism

The genetic relationship among the isolates was determined by fingerprinting using the commercial kit f-AFLP Microbial fingerprinting (Applera; Foster City, California) according to the manufacturer's instructions using as primers: AFLP primer MseI-CC, MseI-CG and AFLP FAM primer EcoRI-O. The f-AFLP reactions were loaded and run on the ABI 310 DNA genetic analyzer (Applera). Each f-AFLP reaction was analysed using Genescan software and Genographer program version 1.6.0 (kindly provided by James J. Benham, http://hordeum.oscs.montana.edu/genographer; Copyright C 1999 Montana State University). Cluster analysis was performed using the unweighted pair group method with the averages algorithm (UPGMA) [18]. The percentage similarity between patterns was calculated using the Dice correlation coefficient [19].

## Results

### Patients and isolates

A total of 17,418 blood samples were screened in 2007, 14,195 from the transplant haematology ward and 3223 from ICU patients (Table 1). For the transplant haematology samples, 1959 were blood culture-positive and of these, 1383 (70.6%) yielded CoNS. Similarly, CoNS were present in 47.3% (226/478) of culture-positive blood samples from the ICU. Overall, using criteria defined in the methods section, 92 out of 1609 CoNS positive blood cultures (5.7%) were considered to be associated with infection. Particularly in the transplant haematology ward 62 out of 1386 (4%) CoNS positive blood cultures caused a BSI while in ICU, the BSI were 30 out of 226 CoNS isolates (13%). In total, 87 CoNS isolates (69 *S. epidermidis*, 18 *S. haemolyticus*) displayed a reduced susceptibility to glycopeptides, a frequency of 5.4%. Of these, 7 and 6 were considered to be associated with infection in patients from the transplant haematology ward and ICU, respectively. Infections caused by CoNS with reduced susceptibility to glycopeptides represent 11% and 20% of all CoNS related BSI in haematology ward and ICU respectively.

**Table 1:** Results of blood culture screening.

	Blood cultures screened (no. positive)	Blood cultures yielding CoNS		Strains with reduced susceptibility to glycopeptides	
		Total	Associated with infection	Total	Associated with infection
Transplant haematology ward	14,195 (1959)	1383	62	76	7
ICU	3223 (478)	226	30	11	6

Clinical data for patients infected by CoNS with reduced susceptibility to glycopeptides are presented in Tables 2 and 3. For transplant haematology patients, all had a central vein device and were bone marrow transplant recipients (Table 2). Not all patients met the full criteria for catheter related blood stream infection (CR-BSI). Nevertheless the infective event was classified as a blood stream infection in the database. For all patients a combination antimicrobial therapy was established according to the therapy protocols of the ward. In all cases microbiology eradication was reached. For ICU patients, all were being treated for one other infection at the time of blood-culture, and all had a central vein device. In patient 9 it was not possible to define whether the BSI was catheter related because the patient died before completing the diagnosis. In ICU the patients were treated with linezolid according to susceptibility test. In all cases, except for patient 9 who was not treated, microbiological eradication was obtained. Overall, only 2 patients (patients 2 and 3, both from the transplant haematology ward) had recorded previous exposure to glycopeptides.

**Table 2:** Characteristics of patients from transplant haematology ward infected with CoNS displaying reduced susceptibility to glycopeptides

Pat.	Age	Underlying disease	Antimicrobial prophylaxis	**Neutrophyl count/mm**^3^	Days from transplantation	Immuno-suppression	Therapy	Catheter removal	CR-BSI	Micro-biological eradication
1	29	ALL	no	2300	43	ciclosporin	linezolid-pip/tazo-amika	no	?	yes
2	40	MM	no	1380	128	ciclosporin	teicoplanin-pip/tazo-amika	no	?	yes
3	34	AML	levofloxacin	10	9	no	linezolid-pip/tazo-amika	yes	yes	yes
4	20	HD	no	6200	3	no	teicoplanin-pip/tazo-amika	no	?	yes
5	52	NHL	levofloxacin	10	5	no	teicoplanin-pip/tazo-amika	yes	yes	yes
6	54	ALL	no	1000	3	ciclosporin	teicoplanin	yes	yes	yes
7	58	MM	no	3700	30	no	teicoplanin-pip/tazo-amika	yes	yes	yes

ALL = acute lymphocitic leukaemia; AML = acute myeloid leukaemia; MM = multiple myeloma; HD = Hodgkin's disease; NHL = non-Hodgkin's lymphoma; MUD = matched unrelated donor; pip/tazo = piperacillin/tazobactam; amika = amikacin; CR-BSI = catheter related blood stream infection; ? = not confirmed

**Table 3:** Characteristics of patients from ICU infected with CoNS displaying reduced susceptibility to glycopeptides.

Patient	Age	Diagnosis on admission	Therapy on day of blood cultures	CR-BSI	Catheter removal	Treatment	Micro-biological eradication
8	59	*Pseudomonas aeruginosa *pneumonia	piperacillin/tazobactam + claritromicin + teicoplanin	yes	yes	linezolid	yes
9	83	Abscess of psoas	piperacillin/tazobactam + metronidazole + teicoplanin	?	no	none	?
10	53	Post neurosurgery cerebral abscess	meropenem + teicoplanin	yes	yes	linezolid	yes
11	59	MDR *P. aeruginosa *pneumonia	meropenem	?	no	linezolid	yes
12	85	*Acinetobacter baumannii *pneumonia	colimicin	yes	yes	linezolid	yes
13	66	Sepsis from infected flebitis in multiple trauma patient	fluconazole	yes	yes	linezolid	yes

CR BSI = catheter related blood stream infection; ? = not confirmed

The majority of isolates (11/13) were identified as *S. epidermidis*, with the remaining two being *S. haemolyticus*, both of which were isolated from transplant haematology ward patients (Table 4). All isolates demonstrated reduced sensitivity to vancomycin (MIC ≥ 2 μg/mL) whereas 7 isolates demonstrated reduced sensitivity to teicoplanin (MIC ≥ 8 μg/mL). The MLSB phenotype, and resistance to oxacillin (MIC ≥ 4 μg/mL) were widespread, each being found in 10 (77%) isolates. All strains remained sensitive to daptomycin, tigecycline and linezolid (Table 4). All strains were also able to grow on Vanco-screen agar according to CLSI algorithm. This means that subpopulations in each strain grow in the presence of ≥ 6 mg/L of vancomycin. Accordingly these strains display heteroresistance and may be associated with therapeutic failure.

**Table 4:** Characteristics of CoNS clinical isolates associated with infection.

Pat	Species	MIC VAN	MIC TEIC	MLSB phenoptype	MIC OXA	MIC DAP	MIC TIG	MIC LZD
Transplant haematology ward isolates								

1	*S. epidermidis*	2	2	yes	4	0.023	0.047	2
2	*S. epidermidis*	2	4	yes	0.5	0.047	0.047	1
3	*S. epidermidis*	2	4	yes	4	0.094	0.064	1
4	*S. haemolyticus*	2	8	yes	4	0.125	0.047	2
5	*S. epidermidis*	2	4	no	0.25	0.047	0.032	1
6	*S. haemolyticus*	2	8	yes	4	0.064	0.047	2
7	*S. epidermidis*	4	16	no	0.25	0.125	0.047	2

ICU isolates								

8	*S. epidermidis*	2	4	yes	4	0.023	0.047	1
9	*S. epidermidis*	2	16	yes	4	0.047	0.047	2
10	*S. epidermidis*	4	≥ 32	no	4	0.094	0.064	1
11	*S. epidermidis*	2	16	yes	4	0.125	0.047	2
12	*S. epidermidis*	2	16	yes	4	0.064	0.047	1
13	*S. epidermidis*	2	4	yes	4	0.047	0.032	2

MIC values are presented as μg/mL. VAN = vancomycin; TEIC = teicoplanin; MLSB = resistance to macrolides, lincosamides and type B streptogramins; OXA = oxacillin; DAP = daptomycin; TIG = tigecycline; LZD = linezolid.

The ratio of *S. epidermidis *to *S. haemolyticus *in the strains associated with infection was 11/2 = 5.5. Overall, including resistant CoNS considered to be contaminants, the ratio *S. epidermidis *to *S. haemolyticus *was 69/18 = 3.8

**Figure 1:**
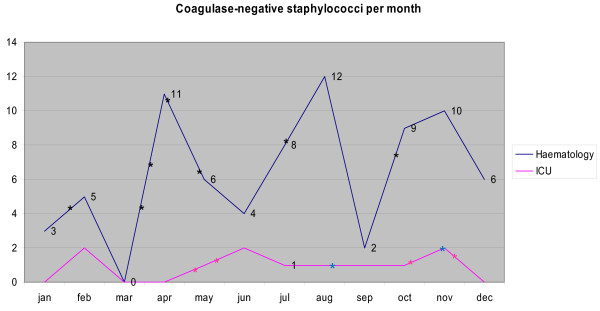
**Isolation frequency by month of CoNS strains with reduced susceptibility to glycopeptides in 2007**. Data are presented for all isolates; the 13 isolates deemed to be associated with infection are indicated by asterisks. Blue asterisks indicate clonal relationship.

There was no obvious trend in isolation of CoNS with reduced sensitivity to glycopeptides across the year of study (Fig. 1).

### Genomic analysis

**Figure 2:**
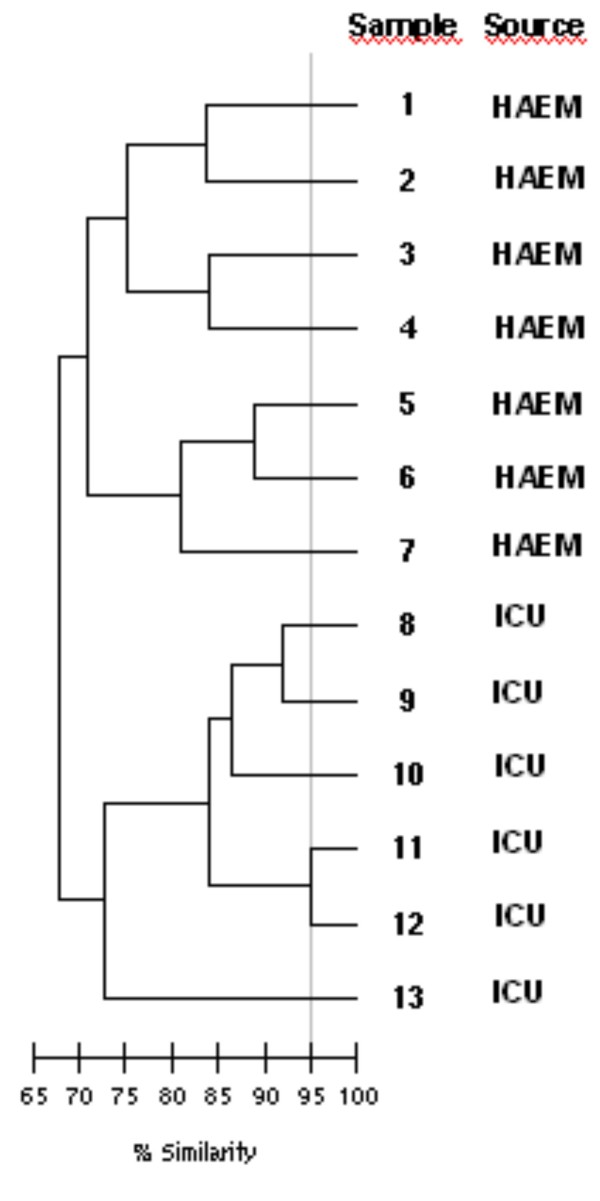
**f-AFLP analysis of the genetic relatedness of CoNS isolates with reduced susceptibility to glycopeptides**.

As shown by f-AFLP profile analysis (Fig. 2), the genetic relatedness among the 13 isolates was low (overall similarity <65%). Cluster analysis revealed two clusters corresponding to transplant haematology ward isolates (overall similarity approximately 70%) and ICU isolates (overall similarity 73%). In the ICU-cluster, two isolates (from patients 11 and 12) had a similarity of 95% and were clonally related. The isolate from patient 11 was retrieved in the month of august while the CoNS from patient 12 was isolated in the month of November. The two patients were admitted and confined in bed in different periods.

## Discussion

All the patients were at increased risk for the development of CoNS infections for different reasons. Patients from the transplant haematology ward were neutropenic or were receiving immunosuppressant drugs, while patients from the ICU were critically ill and were being treated with broad spectrum antibiotics for different infections. Importantly, they all had a central vein catheter. One observation of concern is that only two of the thirteen patients had previous exposure to glycopeptides. This is in contrast to other reports describing the selection of resistance after exposure to vancomycin or teicoplanin in CoNS [8,20]. In the current study, the great number of CoNS isolated from blood were considered to be contaminants, particularly in the haematology ward where blood cultures are drawn for routine for surveillance other than to diagnose infection. In this ward therapy protocols schedule an association of antimicrobials on signs of infections. This may lead to underestimate the frequency of CR-BSI interfering with cultures. In ICU blood cultures are drawn on signs of infection, so the number of clinically relevant CoNS out of the total amount of blood cultures is higher, although the level of contamination is also high. It can be very difficult to draw blood from venipuncture in haematology or critically ill patients, so a certain level of contamination is probably unavoidable, but our data impose to be more strict in sending in blood for cultures in order to limit false positive cultures that may lead to overtreatment.

Our data support the idea that CoNS with reduced susceptibility to glycopeptides are endemic in these wards and are maybe resident on skin and nares of personnel and patients. Indeed, genomic analysis revealed that different strains were responsible for most infections. Only two isolates displayed a clonal relationship. As a direct contact between the two patients was excluded, ward or personnel contamination should be implicated. Nevertheless, as far as we are aware, there is no indication for screening and decontamination of CoNS in patients and personnel from critical areas such as the haematology ward and the ICU. This could be an issue for further studies.

After this study, the actual therapy strategy in the two wards is to start empirical treatment with glycopeptides if CR-BSI is suspected and promptly shift to a different drug according to susceptibility test if the diagnosis is confirmed or if treatment fails. Nevertheless if the signs of infection are severe or if clinical conditions do not allow to wait, therapy with linezolid is started and eventually de-escalated on sensitivity report.

The majority of isolates were *S. epidermidis *and this species was slightly more frequently associated with BSI than *S. haemolyticus*. It was widely accepted that *S. haemoyiticus *is uniquely predisposed among CoNS to develop glycopeptides resistance as this was the first CoNS species in which vancomycin and teicoplanin resistance was identified [21]. Nowadays *S. epidermidis *is often reported to be a nosocomial pathogen bearing multi-resistance [22-24]. Moreover there are many studies suggesting the possible relationship between methicillin resistance and reduced susceptibility to glycopeptides in CoNS [9,25,26].

Not all clinical isolates demonstrated MICs for vancomycin and teicoplanin in the intermediate susceptibility range, but they were all shifted towards the cut-off level. Moreover, these isolates were also able to grow on Vanco-screen agar. It has been previously shown that staphylococci able to grow on Vanco-screen agar may display hetero-resistance to glycopeptides [27]. Heteroresistance was first defined as the presence of >10^-6^stable cell subpopulations of a strain that is apparently susceptible to vancomycin on the basis of conventional criteria, but for which the vancomycin MIC for the subpopulation of cells is greater than or equal to 8 mg/liter [12]. Since 2006 CLSI breakpoints have changed, actual definition might be that although the MIC for the entire population is within the range of susceptibility according to the CLSI criteria, subpopulations grow in the presence of ≥ 4 mg/L of vancomycin. The hetero-resistant phenotype may be associated with treatment failure and/or may be precursor of glycopeptide resistance and should be considered in both empirical and rational therapy decisions.

In our series, CoNS remained homogeneously susceptible *in vitro *to linezolid, tigecycline and daptomycin. Of these three drugs only daptomycin is suitable for the treatment of BSI, given its pharmacokinetic profile. Linezolid was used off label to treat most patients. The use of linezolid for the treatment of catheter related Gram positive blood stream infections is still a matter of discussion, as there have been suggestions that it is associated with a higher mortality rate depending on the type of infection [28]. Nevertheless, in a recent cohort study, empiric therapy with linezolid was associated with greater survival and more successful microbiological eradication compared to standard therapy in the treatment of methicillin-resistant *S. aureus *bacteremia [29]. Further, in a pharmacoeconomic evaluation study linezolid was considered more efficient than teicoplanin in the treatment of Gram-positive bacteremia [30].

Tigecycline is not registered for the treatment of bacteremia, and its use should be limited to real clinical necessity to preserve its activity against multi-resistant Gram negative bacteria. Clinical efficacy of daptomycin has been demonstrated in patients with *S. aureus *bacteremia [31]. To our knowledge no clinical data exist on daptomycin efficacy against CoNS with elevated MICs for glycopeptides. A remarkable characteristic related to low-level resistance to glycopeptides in CoNS is cell wall thickness [27]. Due to its mechanism of action, this characteristic may interfere with daptomycin also, at least in clinical setting or subsequent to extensive use.

## Conclusion

In conclusion, although still relatively infrequent, multiresistant CoNS with reduced susceptibility to glycopeptides are emerging pathogens of clinical concern and should be kept in mind both in empirical and rational therapy of CR-BSI. Surveillance by antibiotyping with attention to multi-resistant profile is mandatory and warning to clinicians is also necessary. Genotyping analysis are useful to discern an epidemic outbreak, which should lead to the implementation of behavioural modifications and, potentially, decontamination may be considered.

## Abbreviations

CLSI: Clinical and Laboratory Standards Institute; CoNS: Coagulase Negative Staphylococci; BSI: Blood stream infection; CR-BSI: Catheter related blood stream infection; EUCAST: European Committee for Antimicrobial Susceptibility testing; f-AFLP: Amplified Fragment Length Polymorphism; ICU: Intensive Care Unit; MIC: Minimum Inhibiotory Concentration; MRSA: Methicillin-resistant *Stapylococcus aureus*; MDR: Multidrug-resistant.

## Competing interests

The authors declare that they have no competing interests.

## Authors' contributions

SN, CF, MF, GPT, AB and FL contributed to the conception, review of the studies and data analysis. SN and CF are also involved in drafting the manuscript. SM MCB, AB, LC, GB, MC and RC contributed in acquisition and interpretation of data. SM and MBC also performed antimicrobial suceptibility tests. MF also performed genetic analysis. All authors approved the final version of the manuscript

## Pre-publication history

The pre-publication history for this paper can be accessed here:

http://www.biomedcentral.com/1471-2334/9/83/prepub
